# Impact of Chronic Kidney Disease on Severity and Mortality in COVID-19 Patients: A Systematic Review and Meta-analysis

**DOI:** 10.7759/cureus.14279

**Published:** 2021-04-03

**Authors:** Trishala Menon, Saad Abdul Quddus Gandhi, Warisha Tariq, Rohit Sharma, Sundus Sardar, Abdullah Mohammad Arshad, Ramesh Adhikari, Fateen Ata, Saurabh Kataria, Romil Singh

**Affiliations:** 1 Family Medicine, Wheeling Hospital, Wheeling, USA; 2 Internal Medicine, Liaquat University of Medical and Health Sciences, Hyderabad, PAK; 3 Internal Medicine, Isra University Hospital, Hyderabad, PAK; 4 Internal Medicine, Hamad Medical Corporation, Doha, QAT; 5 Hospital Medicine, Franciscan Health, Lafayette, USA; 6 Geriatrics, Brown University, Providence, USA; 7 Neurology and Neurocritical Care, University of Missouri Health Care, Columbia, USA; 8 Neurology, West Virginia University, Morgantown, USA; 9 Critical Care, Mayo Clinic, Rochester, USA

**Keywords:** ckd, covid-19, sars-cov-2, chronic renal disease, prognosis

## Abstract

Coronavirus disease 19 (COVID-19) has affected over 180 countries, resulting in global mass death. It has been reported that patients with underlying disease are more likely to contract the disease and become critically ill. The impact of chronic kidney disease (CKD) on the severity of COVID-19 has been underlined in the literature. In this analysis, we have provided evidence of an association between CKD and COVID-19. We followed the PRISMA protocol and conducted a literature search using Google Scholar, EMBASE, PubMed, and Clinical trail.gov. The initial search yielded 2102 articles. We included 20 cohorts based on inclusion criteria reporting an association between CKD and COVID-19 after excluding irrelevant articles, including review articles and duplicates. We conducted pooled prevalence of CKD and meta-analysis to estimate the odds ratio (OR), 95% confidence interval (CI) using Cochrane RevMan (version 5.4, Copenhagen: The Nordic Cochrane Centre, The Cochrane Collaboration), and R programming language version 4.16-2 (University of Auckland, New Zealand). Our study involved 4350 patients from different countries, and 212 (4.9%) patients had CKD. Among 20 cohorts, 57.27% were male with a median age of 55.5 years. Eight hundred sixty-six patients developed severe COVID-19, and out of which, 39 (4.5%) were CKD patients. CKD patients had a significantly increased risk of severe disease as compared to non-CKD patients with a pooled OR of 2.15 (95% CI 1.16-4.01) (I^2^=41; *p*=0.02). Out of 443 COIVD-19 patients who died, 85 patients had CKD, with a prevalence of 19.18%. CKD patients had an increased risk of death as compared to non-CKD patients with a pooled OR of 5.58 (95% CI 3.27-9.54) (I^2^=0; *p*<0.00001). CKD is manifested as a common underlying disease in COVID-19 patients who had a worse prognosis, including mortality.

## Introduction

The newly discovered severe acute respiratory syndrome 2 (SARS-CoV-2), the causative agent of coronavirus disease 19 (COVID-19), has spread rapidly throughout the globe and the World Health Organization (WHO) declared a pandemic in late December 2019. In COVID-19, the patient manifests with a typical acute respiratory illness such as fever, dyspnea, cough, and flu-like symptoms [1,2]. However, COVID-19 can involve multiple organs of the body, including the liver, kidney, heart, and brain. The clinical course of the disease is highly variable and unpredictable, ranging from a flu-like illness to multi-organ failure and death [3].

COVID-19 can affect patients of all ages. However, patients with underlying diseases such as chronic kidney disease (CKD), diabetes, cancer, heart conditions, and chronic obstructive pulmonary disease are at high risk of contracting the disease. The severity of illness and mortality also depend on age and comorbidities [4]. Several studies have shown an association between CKD and COVID-19. CKD of any stage, especially in patients with dialysis, increases the risk of severe disease and mortality [5].

Several meta-analyses have reported the impact of diabetes, heart conditions, COPD, and the outcome and prognosis of COVID-19 patients. However, underlined data on CKD impact on COVID 19 severity and mortality is limited. In the last few months, several studies have been published on the association between end-stage renal disease and SARS-CoV-2 [6]. In this analysis, we have accumulated evidence to provide an association between CKD and the clinical prognosis of COVID-19.

## Materials and methods

Data source and searches

**Figure 1:**
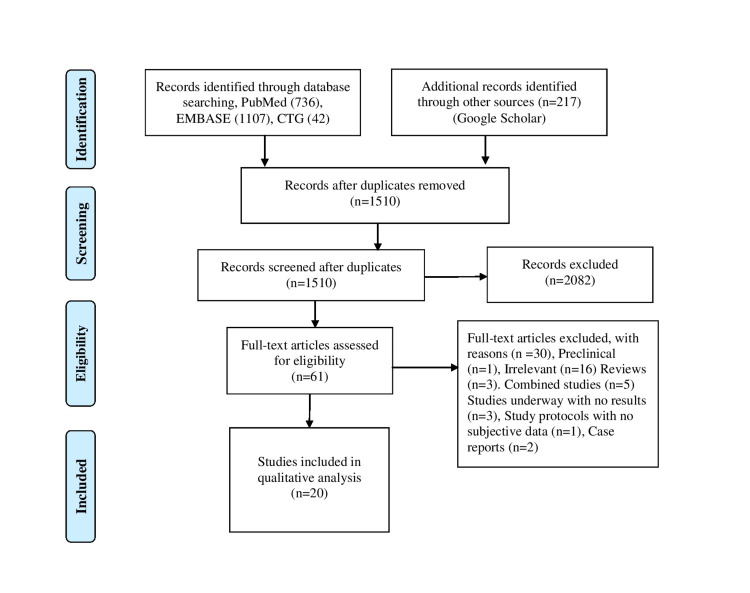
PRISMA flow diagram.

We conducted our study in accordance with the Preferred Reporting Items for Systematic Review and Meta-analyses (PRISMA) protocol (Figure 1). We performed a comprehensive literature search using four electronic databases (PubMed, Google Scholar, EMBASE, and Clinical trial.gov). We did not limit our study to any geographical area or language; in case English translations were present of the study. We performed a data search using the Medical Subject Headings (Mesh) terms and keywords for “renal insufficiency, chronic” OR “chronic kidney disease” AND “COVID-19” from data inception to November 2020. We all also searched the reference of included articles to ensure that all pertinent studies were identified. We downloaded all citations to Endnote version 8.0 (Thomson ISI ResearchSoft, Boston, MA) for further screening and extraction of relevant data.

Study selection and eligibility criteria

We identified 2102 articles on initial screening. One author screened the relevant titles, and two authors screened the abstracts and full texts for relevance. We excluded case reports, pediatric studies, abstracts, and review articles (Table 1). We also excluded all those studies with particular populations such as malignant patients, heart patients, and patients reporting only acute kidney injury. Initially, we included full-length articles meeting the inclusion criteria based on the study title and screened each study in-depth for inclusion. We selected prospective and retrospective articles reporting data on CKD and COVID-19 related to disease severity and mortality with more generalized population and extractable data. All the articles were assessed for exclusion and inclusion criteria, and the disagreements were resolved through discussion and consensus.

**Table 1:** Characteristics of included studies. CKD: chronic kidney disease, NA: not available.

Author	Year	Study type	Study location	No of patients	Median age (yrs)	Male (%)	CKD patients	Severe COVID-19	Mortality
Cao et al. [7]	2020	Retrospective, single-center	China	102	54	75	4	NA	17
Chen et al. [8]	2020	Single-center, retrospective	China	274	62	62.4	5	NA	113
Mei et al. [9]	2020	Multicenter, retrospective	China	223	72	50.2	83	NA	132
Wang et al. [10]	2020	Single-center, retrospective	China	107	51	53.3	3	36	NA
Yan et al. [11]	2020	Single-center, retrospective	China	193	64	59.1	4	NA	108
Zhou et al. [12]	2020	Multicenter, retrospective	China	191	56	62	8	NA	54
Aggarwal et al. [1]	2020	Single-center, retrospective	USA	16	67	52	6	8	NA
Chen et al. [2]	2020	Single-center, retrospective	China	145	47.5	54.5	3	43	NA
Feng et al. [3]	2020	Single-center, retrospective	China	114	64	62.3	6	20	NA
Hu et al. [13]	2020	Single-center, retrospective	China	323	61	51.4	7	172	NA
Huang et al. [14]	2020	Single-center, prospective	China	41	49	73	4	13	NA
Liu et al. [4]	2020	Multicenter, retrospective	China	620	44.48	52.6	4	53	NA
Qin et al. [15]	2020	Single-center, retrospective	China	452	58	52	10	286	NA
Regina et al. [16]	2020	Retrospective, observational	Switzerland	200	70	60	28	37	NA
Shabrawishi et al. [17]	2020	Single-center, retrospective	Saudi Arabia	150	46.1	60	10	16	NA
Shi et al. [18]	2020	Single-center, retrospective	China	487	46	53.2	7	49	NA
Wan et al. [19]	2020	Single-center, retrospective	China	135	47	53.3	6	40	NA
Yan et al. [20]	2020	Multicenter, retrospective	China	218	42.9	56	4	38	NA
Zhang et al. [21]	2020	Single-center, retrospective	China	221	55	48.9	6	55	NA
Wang et al. [22]	2020	Single-center, retrospective	China	138	56	54.3	4	NA	19

Data extraction and quality evaluation

The standardized extraction sheet was developed for clinical information and relevant data. For each included study, we extracted the following data: author, year, gender, study design, median age, patient number, and CKD patients. Severe disease and mortality in CKD patients were the primary interest in our study. We assessed the quality of studies using the Newcastle-Ottawa Quality Assessment Scale (NOS; Appendices). The potential publication bias and small study effects were evaluated using a funnel plot and minor if there was an approximate symmetrical funnel shape.

Statistical analysis

Our search included 20 studies from different countries. A random effect analysis was conducted for pooled prevalence estimates, pooled odds ratio (OR), 95% confidence intervals (CI) for severe disease, and prognosis of COVID-19 patients in CKD using Cochrane RevMan (version 5.4) and R programming language version 4.16-2. The heterogeneity in the analysis was estimated by the *I^2^* test, which measures the total variation among the studies. We applied a random effect model for moderate heterogeneity, and for the appropriate level of heterogeneity, a fixed-effect model was applied.

## Results

A total of 61 articles out of 2102 articles were assessed for eligibility, and 20 articles were included in the analysis. Our study included 4350 patients from different countries, and 212 (4.9%) patients had CKD. Most of the patients were hospitalized, and we only included those articles reporting an association between CKD and COVID-19 due to the potential overlapping of cohorts in several studies. The patients were considered to have CKD who had a preexisting renal disease or had high serum creatinine before admission, meeting the Kidney Disease's diagnostic criteria: Improving Global Outcomes (KDIGO) guidelines. COVID-19 patients with severe dyspnea, low oxygen saturation (<93%), and more than 50% lung infiltrates within one to two days were included in the definition of severe COVID-9. Patients requiring mechanical ventilation and admission to the intensive care unit admission were also considered severe and critically ill.

**Figure 2:**
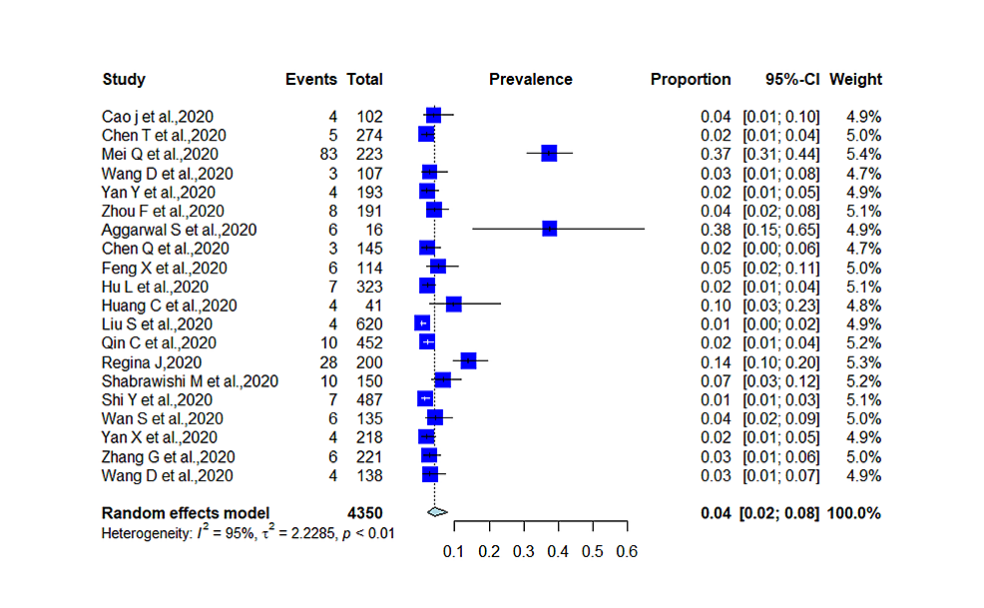
Prevalence of CKD in COVID-19 patients. CKD: chronic kidney disease, COVID-19: coronavirus disease 19.

Among 20 studies, 57.27% were male, and the median age was 55.5 years SD 8.87. Out of 4350 COVID-19 patients, 212 had CKD disease with prevalence of 4% (95% CI 0.02-0.08; *I*^2^=95%; *p *< 0.01; Figure 2).

**Figure 3:**
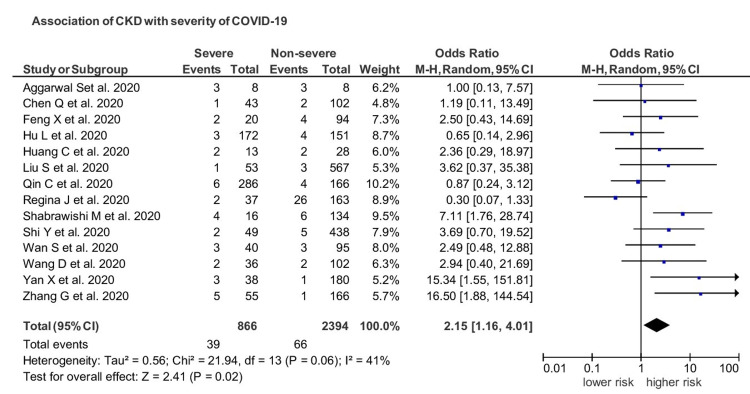
Impact of CKD on the severity of COVID-19. CKD: chronic kidney disease, COVID-19: coronavirus disease 19.

**Figure 4:**
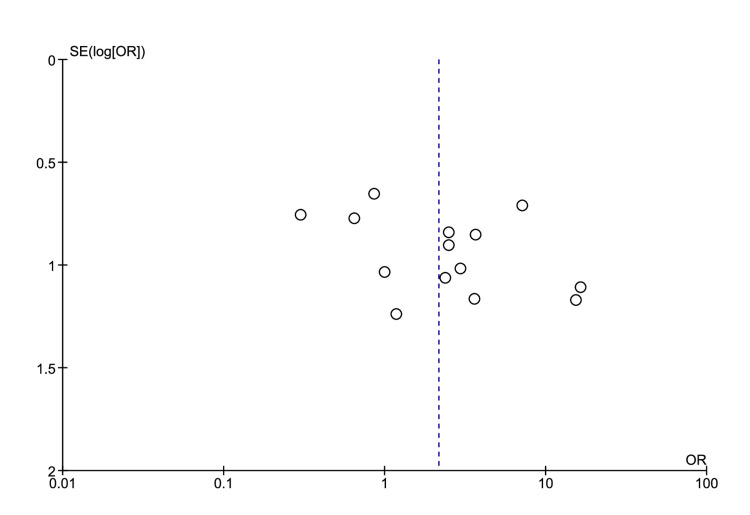
Funnel plot showing an association between CKD and COVID-19. CKD: chronic kidney disease, COVID-19: coronavirus disease 19.

Fourteen studies yielded enough data on the severity of disease in CKD patients. Among 20 cohorts, 866 patients developed severe COVID-19 and out of which 39 (4.5%) were CKD patients. CKD patients had a significantly increased risk of severe disease as compared to non-CKD patients with a pooled OR of 2.15 (95% CI 1.16-4.01; *I*^2^=41; *p*=0.02; Figure 3). The funnel plot revealed an association between CKD and severe disease with no publication bias (Figure 4).

**Figure 5:**
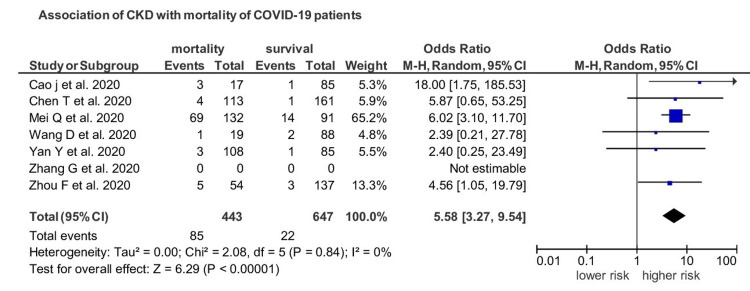
Impact of CKD on prognosis COVID-19 patients. CKD: chronic kidney disease, COVID-19: coronavirus disease 19.

Seven studies provided data on mortality in CKD patients. Out of 443 COIVD-19 patients who died, 85 patients had CKD, with a prevalence of 19.18%. CKD patients had an increased risk of death as compared to non-CKD patients with a pooled OR of 5.58 (95% CI 3.27-9.54; *I*^2^=0; *p*<0.00001; Figure 5).

## Discussion

The population at any age with CKD is at increased risk of severe COVID-19. CKD is one of the most predominant factors for severe SARS-CoV-2 infection throughout the world and is the most common risk factor at any age. It has been reported that one in four high-risk individuals for COVID-19 worldwide has CKD. Thus, removing CKD as a risk factor would reduce the global population percentage at enhanced risk of COVID-19 from 22% to 17%, comparable to 5% of the world population [23]. Recent studies have reported an increased frequency of kidney abnormalities. Li et al. underlined that 34% of infected SARS-CoV-2 patients had massive albuminuria and 63% of patients developed proteinuria, and 27% of patients had elevated BUN [24]. A recent study reported 44% proteinuria, 27% hematuria, and BUN 14.1% in 710 hospitalized patients infected with COVID-19 [25]. Computed tomography of these patients revealed chronic renal changes, including inflammation, edema, and reduced density [25]. Our analysis reported evidence of CKD impact on prognosis and outcome of COVID-19 patients and underlined a significant association of CKD with severe infection with a pooled OR of 2.15 (95% CI 1.16-4.01) (I^2^=41) (*p*=0.02). Regarding the prognosis of the patients, out of 443 COIVD-19 patients who died, 85 patients had CKD, with a prevalence of 19.18%. CKD patients had an increased risk of death than non-CKD patients with a pooled OR of 5.58 (95% CI 3.27-9.54). The results were substantial (*p*<0.00001) with negligible heterogeneity.

Flythe et al. conducted a study to determine the clinical progression of severe COVID-19 in CKD patients and investigated the outcome and clinical prognosis. He reported that CKD patients are more susceptible to severe disease, marked by an intense inflammatory response, thrombosis, and multisystem organ failure [26]. The study revealed that among 4,264 patients, both non-dialysis patients and maintenance dialysis patients had higher hospital mortality rates up to 50%. Patients with underlying CKD had high mortality rates as compared to the patients without preexisting CKD. Patients with dialysis are at increased risk of contracting disease and mortality [26]. Furthermore, another study supported the results by Williamson et al., which revealed that advanced CKD was among those conditions carrying the highest risk of death and remarkably higher than that discussed by all other study factors. This study also revealed that CKD with grades 4-5 was among the top high categories (adjusted hazard ratio: 2.52). The risk of severe disease was even higher than diabetes and asthma. After sensitivity analysis with different populations (adjusted for imputed ethnicity, early censoring, and restricted to those with body mass index), the risk of disease conversed by severe CKD was consistently higher [27]. Another study by Brosetta et al. also reported an association of severe COVID-19 with CKD patients, particularly patients on dialysis [6]. A recent analysis by Wang et al. further revealed the association of CKD with COVID-19 severity and mortality and reported that CKD patients had a remarkably increased risk of severe disease progression (OR: 2.31, 95% CI) and also had increased risk of mortality (OR: 5.11, 95% CI) [28]. Our analysis also has a parallel tendency in reporting the association of CKD with the severity and mortality of COVID-19.

Many suggestions have been explained why CKD morbidity has an increased risk of progression to severe disease or death. CKD patients have a pro-inflammatory state and functional defects in innate and acquired immune cells, resulting in increased vulnerability to infection [5,29]. There is an increased risk of pneumonia and upper respiratory tract infection in CKD patients, which may be a reason for concurrent infection with COVID-19. Comorbidities, especially heart conditions and diabetes, usually coexist with CKD, and these conditions are associated with worse outcomes in COVID-19 patients. A recent cohort of 1,000 patients in CKD patients reported that increased albumin-creatinine ratios and reduced glomerular filtration rate (GFR) were associated with higher infection rate, severity, increased risk of hospitalization, and mortality [6]. CKD is more prevalent in old age than in adults, which justifies the increased burden of disease morbidity and mortality in CKD patients.

This analysis has many limitations. We included only prospective and retrospective cohorts, and any randomized clinical trial was excluded. Residual confounding may exist with observational studies. The individual patient data in many studies were not available; therefore, we could not measure our adjustments. Publication bias is also the limitation as there is a less likely chance of negative study publication. Rapidly emerging literature of COVID-19 also concerns, which may result in a comparatively short follow-up period and presence of ambiguity and limitations in their detailed portrayal. Furthermore, this growing data also provide difficulty in relevant evidence retrieval on the concerned subject. We included only those studies reporting CKD in more than two patients to avoid bias. We have not included the preprint of the studies, as well as abstracts.

The outcome and prognosis of COVID-19 patients are affected by comorbidities, and CKD has a significant role in patient outcomes [26]. Given the extent of the steady relationship between CKD and COVID-19 prognosis and the impact of CKD on survival and outcomes, there is a dire need for comprehensive data on CKD impact on COVID-19 that allows a proper acknowledgment of disease during this pandemic.

## Conclusions

Patients with comorbidities, particularly CKD, have more deteriorating outcomes and worse prognoses, including mortality. There is an emerging body of evidence that CKD significantly impacts severity and mortality in COIVD-19 patients. Therefore, necessary measures should be exercised while monitoring the CKD patients with COVID-19 regardless of other comorbidities and functional status of the patient. CKD association with severe disease and mortality in our study will help the physician to have an essential preparation in CKD patients, which is of supreme importance in preventing and managing the disease. There is a dire need for public health campaign regarding awareness of the disease, its complication in CKD, and reducing the burden of this comorbidity causing death in COVID-19 patients.

## Appendices

**Table 2:** Risk of bias for included studies. Risk of bias assessment: each article was given a rating (low=1, medium=2, high=3) according to the NOS. Discrepancies were resolved by a third reviewer if necessary.

Author	Reviewer 1	Reviewer 2	Reviewer 3
Cao et al. [7]	3	3	N/A
Chen et al. [8]	3	3	N/A
Mei et al. [9]	3	3	N/A
Wang et al. [10]	3	3	N/A
Yan et al. [11]	2	2	N/A
Zhou et al. [12]	3	3	N/A
Aggarwal et al. [1]	3	3	N/A
Chen et al. [2]	3	3	N/A
Feng et al. [3]	2	2	N/A
Hu et al. [13]	3	3	N/A
Huang et al. [14]	3	3	N/A
Liu et al. [4]	3	3	N/A
Qin et al. [15]	3	3	N/A
Regina et al. [16]	2	2	N/A
Shabrawishi et al. [17]	2	2	N/A
Shi et al. [18]	2	2	N/A
Wan et al. [19]	3	3	N/A
Yan et al. [20]	3	3	N/A
Zhang et al. [21]	3	3	N/A
Wang et al. [22]	3	2	2
